# Poly(3-hexylthiophene) Grafting and Molecular Dilution: Study of a Class of Conjugated Graft Copolymers

**DOI:** 10.3390/polym11020205

**Published:** 2019-01-24

**Authors:** Tomasz Jarosz, Kinga Kepska, Przemyslaw Ledwon, Marcin Procek, Wojciech Domagala, Agnieszka Stolarczyk

**Affiliations:** 1 Department of Physical Chemistry and Technology of Polymers, Silesian University of Technology, 9 Strzody Street, 44-100 Gliwice, Poland; tomasz.jarosz@polsl.pl (T.J.); kinga.kepska@polsl.pl (K.K.); przemyslaw.ledwon@polsl.pl (P.L.); wojciech.domagala@polsl.pl (W.D.); 2 Department of Inorganic Chemistry, Analytical Chemistry and Electrochemistry, Silesian University of Technology, 6 Krzywoustego Street, 44-100 Gliwice, Poland; 3 Department of Optoelectronics, Silesian University of Technology, 2 Krzywoustego Street, 44-100 Gliwice, Poland; marcin.procek@polsl.pl

**Keywords:** poly(3-hexylthiophene), graft copolymers, molecular dilution, functionalized conducting polymers

## Abstract

A type of graft copolymer based on polysiloxane and regioregular poly(3-hexylthiophene) (P3HT) has been synthesised and its properties have been studied alongside those of its parent conjugated polymer—regioregular P3HT. Electrochemical analysis has revealed more significant changes in conformation of the copolymer film than was observed for P3HT. UV-Vis-NIR spectroelectrochemical investigation provided evidence of improved doping reversibility of the copolymer, despite its marginally increased band gap, as also confirmed by electroconductometric analysis. Evidence has been shown, indicating that polaron mobilities in both P3HT and the copolymer are higher than those of bipolaronic charge carriers, even though both systems exhibit standard doping/dedoping patterns. The grafted copolymer was tested in bulk heterojunction solar cells. Preliminary studies show a great potential of these polymers for application in photovoltaics. Power conversion efficiency of up to 2.46% was achieved despite the dilution of the P3HT chains in the copolymer.

## 1. Introduction

Organic semiconductors, with their flexibility and cost-efficiency, are an ever-evolving alternative to silicon-based optoelectronics. The labours of numerous researchers worldwide have brought about impressive advances in organic photovoltaic cells (OPVs), light-emitting diodes (OLEDs), and field-effect transistors (OFETs), and the reports of their performance are nothing but promising. The evolution of the field is ensured by constant development of new conjugated materials; however, it is the formulation of new concepts (donor–acceptor copolymers, bulk heterojunction) that engenders breakthroughs.

Organic optoelectronic devices typically use indium–tin oxide (ITO)/glass or ITO/quartz as one of the electrodes, with the other one typically being metallic. Out of many systems interacting with such oxide-based substrates, siloxanes are prime candidates due to their high affinity for such materials and cost-efficiency. Therefore, grafting conjugated polymers onto siloxane or polysiloxane scaffolds may be sufficient to achieve improved adhesion to oxide-based electrodes [1]. Furthermore, organosilanes can be made to self-organise into layered lamellar structures, whose arrangement may be controlled to a significant extent [2].

Poly(methylhydrosiloxane) is a very convenient substrate for designing such tailor-made architectures. The reactivity of the silane (Si–H) bond allows grafting a broad selection of side groups including bulky moieties [3]. Polysiloxanes grafted with various side groups are materials of interest to many research groups. There are publications describing such copolymers and their possible applications depending on diverse properties attributed to grafted fragments [4,5,6,7,8]. However, to the best of our knowledge, there are no reports of polysiloxanes grafted with conjugated polymers tailored for optoelectronic applications in literature.

Among many π-conjugated polymers, poly(3-alkylthiophenes) (P3ATs) are widely studied due to their long conjugation length, relatively high charge mobility [9], stability, solution processability [10], and formation of semi-crystalline structures [11]. Whereas the composition and average molecular weight of the polymer can be easily tuned, control over its regioregularity was achieved with the development of novel polymerisation procedures [12]. The pioneering McCullough method [13] and his later procedures [14] allowed P3ATs with degrees of regularity exceeding 98% to be prepared. What is more, that synthetic path allows in situ end-functionalization with various groups [15]. Such highly regioregular P3ATs have proven to possess conductance far superior to that of their regiorandom analogues [16].

In this work, we continue our earlier investigation [17] into the structure of P3HT graft copolymers, mounting P3HT grafts either standalone or alongside non-conjugated PEG chains on our polysiloxane scaffolds. The choice of P3HT was based on the fact that it is a well-known, hole-transporting system, which could be readily grafted onto a polysiloxane chain in a controlled manner. We have compared the properties of our graft copolymer to its parent system—regioregular poly(3-hexylthiophene) and hereby present the results of our investigations of the electric (via conductometry), electrochemical (via cyclic voltammetry), spectroelectrochemical (via EPR-UV-Vis-NIR spectroelectrochemistry), and colour switching (via time-resolved UV-Vis spectroelectrochemistry) properties of these two systems, along with the results of investigation of early solar cell prototypes, constituting what we earnestly believe to be an early proof of the laid out concept for the novel class of copolymers.

## 2. Materials and Methods

### 2.1. Materials

The 2,5-dibromo-3-hexylthiophene *≥* 97%, TCI (Tokyo, Japan), t-butylmagnesium chloride (2M ether solution) (Sigma Aldrich, St. Louis, MO, USA), dichloro-[1,3-bis(diphenylphosphino)propane]nickel(II) (Ni(dppp)Cl2) (≥97%, Sigma Aldrich), vinylmagnesium bromide (0.7 M solution in tetrahydrofuran, (Acros Organics Geel, Belgium), poly(methylhydrosiloxane) trimethylsilyl terminated (average *M*_n_ ~390—PMHS390—and 1700–3200—PMHS1700/3200, Sigma Aldrich), platinum(0)-1,3-divinyl-1,1,3,3-tetramethyldisiloxane (PTDD, Karstedt’s catalyst) (complex solution in xylene, Pt ~2%, Sigma Aldrich), poly(ethylene glycol) methyl ether methacrylate (PEG) (average Mn 500, Sigma Aldrich), anhydrous toluene (99.8%, Acros Organics) were used as received, without further purification. Anhydrous tetrahydrofuran (99.9%, Acros Organics) was distilled over metallic sodium prior to use. All reactions were conducted under dry nitrogen or argon flow, in oven-dried glassware.

### 2.2. Syntheses (for Detailed Information See Appendix A)

**Scheme 1:**
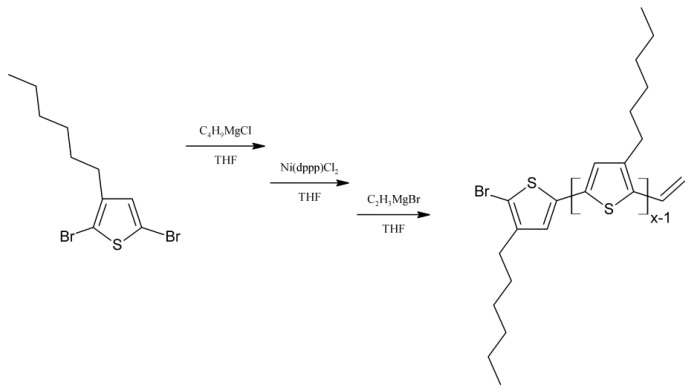
Synthesis scheme of vinyl terminated regioregular poly(3-hexylthiophene) (P3HTvin) via GRIM procedure.

Vinyl terminated regioregular poly(3-hexylthiophene) (P3HT) with fractions of molecular weights of 4000 (hexane fraction) and 10,000 g/mol (chloroform fraction), and dispersities of 1.4 have been synthesised (as determined by size exclusion chromatography, SEC) via the McCullough GRIM method [18], with modifications based on the work of Boudouris [19] (Scheme 1).

Crude polymer was purified with sequential Soxhlet extraction with methanol, hexane, and chloroform. Vacuum-dried hexane and chloroform fractions were characterised by means of ^1^H-NMR and SEC analyses and used for further syntheses.

**Scheme 2:**
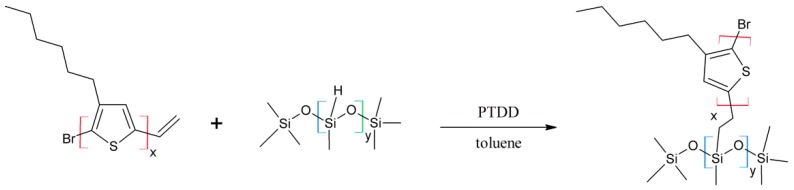
Synthetic route to poly(methylhydrosiloxane)-graft-poly(3-hexylthiophene) (P3HT-Sil).

**Scheme 3:**
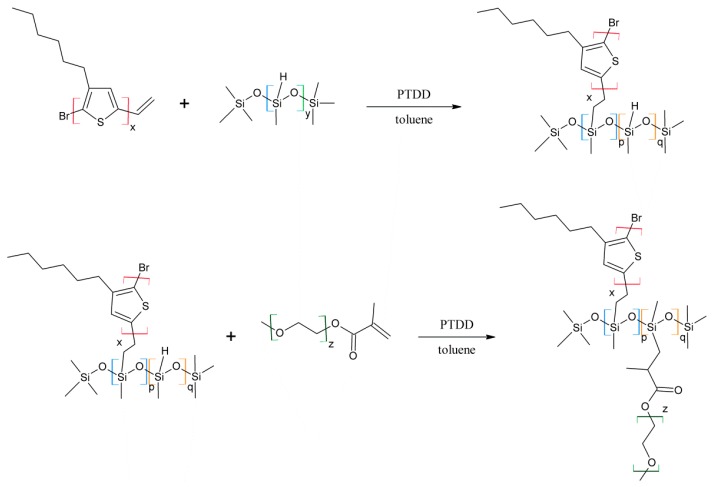
Synthetic route to poly(methylhydrosiloxane)-graft-[poly(3-hexylthiophene); poly(ethylene glycol)]s (P3HT-Sil-PEGs).

Poly(methylsiloxane)-graft-poly(3-hexylthiophene) (**P3HT-Sil**; Scheme 2) and poly(methylsiloxane)-graft-poly(3-hexylthiophene)-graft-poly(ethylene glycol) copolymers (**P3HT-Sil-PEG1**, **2** and **3**; Scheme 3) were prepared via variation of the hydrosilylation reaction, conducted according to method described by Ganicz [3].

Crude products were purified by means of sequential Soxhlet extraction with methanol and chloroform (P3HT-Sil) or methanol, hexane, and chloroform (**P3HT-Sil-PEGs**). The hexane fraction of **P3HT-Sil-PEG1** and chloroform fractions of **P3HT-Sil**, **P3HT-Sil-PEG2**, and **P3HT-Sil-PEG3** were used for spectroelectrochemical investigations. Details of the copolymers are compiled in Table 1.

### 2.3. Molecular Characterisation (for Detailed Information See Appendix A)

^1^H-NMR analysis of products was performed for solutions in CDCl_3_ on a Varian Unity Inova (Palo Alto, CA, USA) spectrometer with a resonance frequency of 300 MHz using TMS as internal standard. IR spectroscopy was carried out on Perkin–Elmer Spectrum Two (Waltham, MA, USA) spectrometer with UATR (Single Reflection Diamond) module. The number average molecular weights and dispersities were determined for THF solutions of sample concentration 1 mg/cm^3^ using a size-exclusion (SEC) 1100 Agilent (Santa Clara, CA, USA) 1260 Infinity isocratic chromatograph with a differential refractometric MDS RI Detector (USA). The molecular weight obtained by SEC was based on calibration with linear polystyrene standards (580–300,000 g/mol).

### 2.4. Electrochemical, Spectroelectrochemical, and Conductometric Meausrements

Solutions for electrochemical and spectroelectrochemical investigation were prepared by dissolving the polymer sample in chloroform (Sigma-Aldrich, CHROMASOLV, >99.9%, HPLC grade). Thin solid films on platinum or ITO/quartz working electrodes were prepared through drop-casting the polymer solution, typically at a concentration of 1 mg/cm^3^ onto an electrode. All films were investigated in a 0.1M solution of tetrabutylammonium hexafluorophosphate (Sigma-Aldrich, >99.0%, electrochemical analysis grade) in acetonitrile (Sigma-Aldrich, CHROMASOLV, >99.9%, HPLC grade), acting as a supporting electrolyte.

AFM microscopy was performed on an NT-MDTN_ITEGRA Prima platform (NT-MDT, Moscow, Russia) using the intermittent contact mode. The images of thin solid films prepared through drop-casting on ITO/quartz electrode were obtained at a resonance frequency equal to 136.281 kHz, both for P3HT and P3HT-Sil-PEG1. The HA_NC (NT-MDT) tip was used in those measurements.

Electrochemical investigations were performed using a standard three-electrode cell, with a Pt or indium–tin oxide (ITO) working electrode, an Ag pseudo-reference electrode, and a Pt coil counter electrode. Measurements were carried out on a Metrohm-Autolab PGSTAT100N (Herisau, Switzerland) potentiostat. Prior to measurement, each investigated sample was purged with inert gas, while the same gas was being passed through the electrochemical cell during the measurement.

UV-Vis-NIR spectroelectrochemical experiments were carried out in a 2 mm Hellma QS cuvette, equipped with an ITO/glass working electrode, Ag pseudo-reference electrode, and a Pt mesh counter electrode. UV-Vis-NIR spectra were registered using an Ocean Optics (Largo, FL, USA) diode-array spectrometers set (QE65000 and NIRQuest512). In the potential staircasing experiments, UV-Vis-NIR spectra were recorded approximately 100 s after each potential step was applied. Optical contrast values were calculated using transmittance of the polymer films in their most highly doped and undoped states; these values were then averaged across all wavelengths in the spectral range of the spectrometer.

Electron Paramagnetic Resonance (EPR) spectra were acquired using a JEOL JES FA-200 (Tokyo, Japan) X band spectrometer. A capillary spectroelectrochemical cell was used for this purpose, with the same electrode configuration as in the standard electrochemical experiments. Relative spin concentrations for each potential step have been obtained through manual double integration of the first derivative EPR spectrum.

Electroconductometric experiments have been performed using interdigitated array electrodes (Pt path width of 5 μm and path spacing of 5 μm, Dropsens (Herisau, Switzerland), comprising two working electrodes, with a Pt flag auxiliary electrode and an Ag wire pseudo-reference electrode. Measurements were realised in cyclic voltammetric mode, scanning the potential while, maintaining a constant potential offset between of the two working electrodes. All electroconductometric experiments have been conducted in a supporting electrolyte solution that has been initially purged with argon, but was exposed to air during the experiment. This was done in order to investigate the polymer films in conditions, as similar as possible to those, in which we expect the polymers to be applied.

Applied potentials in all of the electrochemical experiments were calibrated versus the ferrocene/ferrocinium redox couple as presented. In the case of spectroelectrochemical experiments, the applied potentials refer to the silver pseudo-reference electrode system. The silver pseudo-reference electrode was calibrated against ferrocene/ferrocinium redox couple, whose recorded potential versus this electrode was constant, amounting to +0.46V.

### 2.5. Solar Cells Fabrication and Characteristic

Solar cells with the structure: ITO/PEDOT:PSS/active layer/Al, were prepared using ITO Glass Substrates (Ossila, 6 pixels). PEDOT:PSS (M121, Ossila) films were fabricated by spin coating with 5000 rpm and drying at 140 °C for 10 min in air. Active layers were obtained from chlorobenzene (99+, Acros Organics) solutions with P3HT-Sil-PEG3/PC_61_BM (2:1) or P3HT/PC_61_BM (2:1) with a total concentration of 25 mg/ml, by spin coating with 2000 rpm and then drying at 120 °C for 10 min. IV-curves of photovoltaic devices were measured by PV Test Solutions Solar Simulator and Keithley 2400 (Tektronix, Inc., Beaverton, OR, USA).

## 3. Results and Discussion

### 3.1. Material Identification

**Scheme A2:**
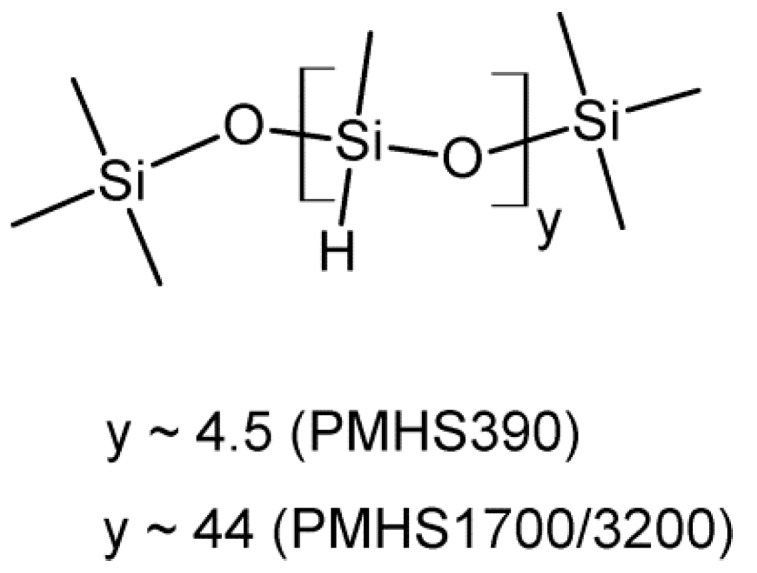
Structure of poly(methylhydrosiloxane) trimethylsilyl terminated (PMHS).

**Figure A1:**
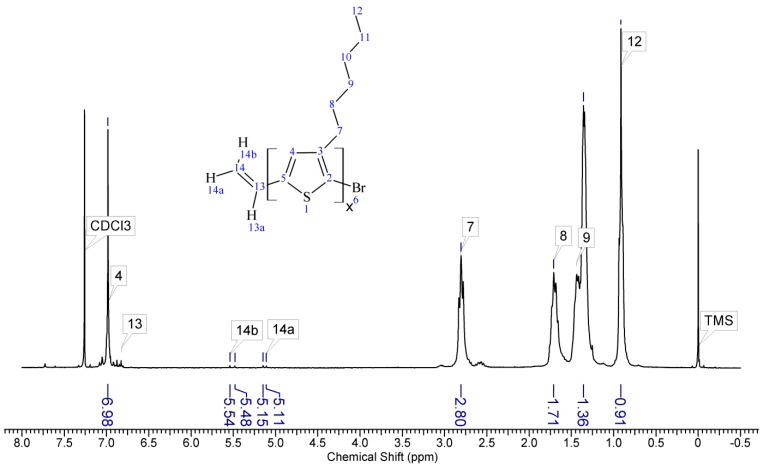
Spectrum of vinyl terminated regioregular P3HT (**P3HTvin**).

**Figure A2:**
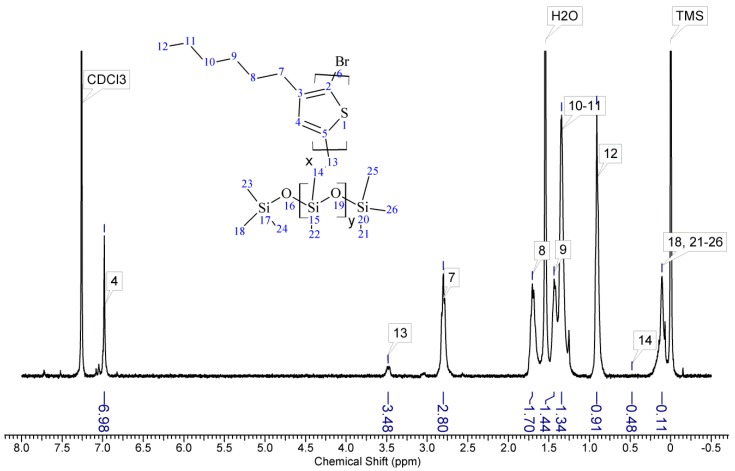
Spectrum of poly(methylhydrosiloxane)-graft-poly(3-hexylthiophene) (composed ofchloroform fraction of vinyl terminated poly(3-hexylthiophene), poly(methylhydrosiloxane) average *M*_n_ ≈ 390; chloroform fraction of graft copolymer) (**P3HT-Sil**).

**Figure A3:**
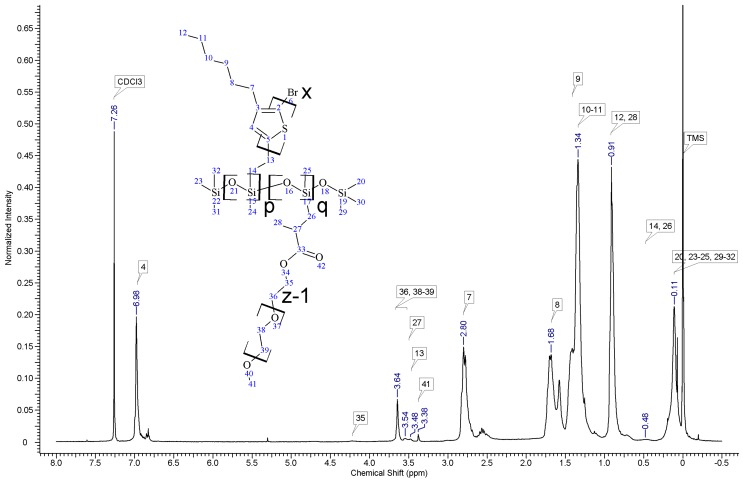
Spectrum of poly(methylhydrosiloxane)-graft-[poly(3-hexylthiophene); poly(ethylene glycol)] (composed of hexane fraction of vinyl terminated poly(3-hexylthiophene),poly(methylhydrosiloxane) average *M*_n_ ≈ 390; hexane fraction of graft copolymer) (**P3HT-Sil-PEG1**).

**Figure A4:**
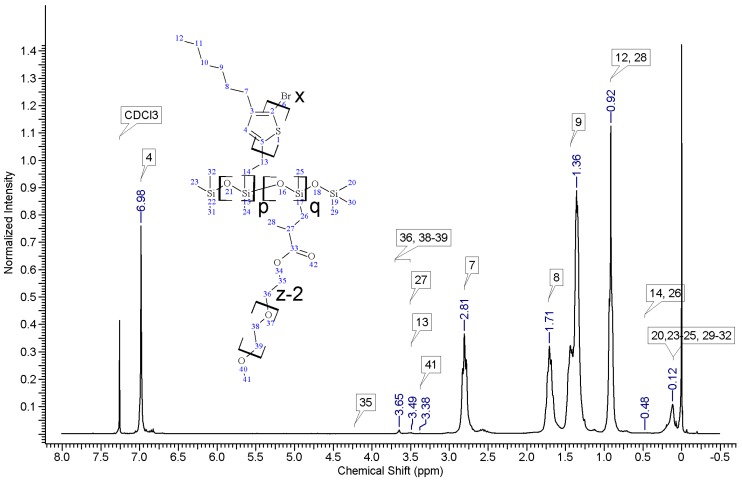
Spectrum of poly(methylsiloxane)-graft-[poly(3-hexylthiophene); poly(ethylene glycol)] (composed of chloroform fraction of vinyl terminated poly(3-hexylthiophene), poly(methylhydrosiloxane) average *M*_n_ ≈ 390; chloroform fraction of graft copolymer) (**P3HT-Sil-PEG2**).

**Figure A5:**
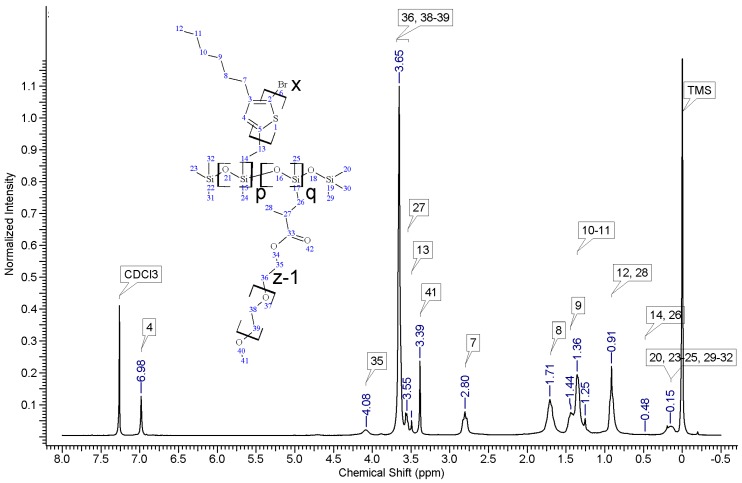
Spectrum of poly(methylsiloxane)-graft-[poly(3-hexylthiophene); poly(ethylene glycol) (composed of chloroform fraction of vinyl terminated poly(3-hexylthiophene), poly(methylhydrosiloxane) average *M*_n_ ≈ 1700–2300; chloroform fraction of graft copolymer) (**P3HT-Sil-PEG3**).

Regioregular poly(3-hexylthiophene) functionalised with terminal vinyl group was synthesised via the GRIM method with in situ end group attaching. Polysiloxane-graft-(P3HT; PEG) samples were prepared via a variation of the hydrosilylation reaction, in the course of a facile one-pot synthesis. In all syntheses, an excess of PMHS over P3HT was used. The structure of the obtained products was confirmed by comparing the ^1^HNMR spectra of reactants and the final product. On the spectrum of the copolymer, the disappearance of the signal of protons originating from the vinyl group at δ 5.13 and 5.51 ppm [18] and the disappearance of the proton signal of hydrogen atom attached to the silicon atom in the main chain (4.67–4.72 ppm) is observed. Comparative analysis of ^1^H-NMR spectra (see Appendix A) indicate that grafting on poly(methylhydrosiloxane) backbones has occurred in each case. The vinyl terminated P3HT spectrum clearly shows the signals of the geminal protons in vinyl terminal group: two doublets at δ 5.51 (H14b, J_H14b-H13a_ = 17.8 Hz) and 5.13 ppm (H14a, J_H14a-H13a_ = 11.4 Hz) (Figure A1 in Appendix A). The signal of the third proton of vinyl group, adjacent to thiophene ring (H13a, ddδ6.84 ppm) overlaps with the group of aromatic protons signals. There are no vinyl proton signals on any copolymer spectrum, but a new signal at δ3.48 ppm appears, derived from the protons of the –CH_2_– group adjacent to the aromatic ring originating from the vinyl group being transformed in the course of grafting vinyl terminated P3HT onto poly(methylhydrosiloxane) backbone (Figure A2, Figure A3, Figure A4 and Figure A5, Scheme A2 in Appendix A). A broad signal of the Si–CH_3_ protons originating from the polysiloxane chains appears on the spectra of all copolymers at δ0.10–0.25 ppm and a broad signal of new Si–CH_2_ groups at about 0.48–0.50 ppm arises, while the Si–H proton signal at δ4.67–4.72 ppm declines. Also. the signal at δ0.10–0.25 ppm is broadened and significantly different comparing to original signal on PMHS spectrum. This implies changes arising from alteration of Si–CH_3_ protons surrounding resulting from the grafting. Furthermore, it should be noted that if the P3HTvin and polysiloxane segments were not chemically bonded, the elution of unbound polysiloxane chains during methanol extraction would occur. Simultaneously, the abovementioned δ0.10–0.25 ppm signals of Si–CH_3_ protons would not be present in the hexane and chloroform fractions of the graft copolymers. On all P3HT-Sil-PEGs spectra the signals of O–CH_2_ (δ 3.65–3.55 ppm) and O–CH_3_ protons (δ 3.38 ppm) are present, confirming grafting of polyether co-monomer at polysiloxane backbone.

In all cases of grafting, excess of PMHS was used. During the first step of grafting P3HT chains are attached to the polysiloxane backbone, then in the second step PEG is added to saturate unreacted Si–H groups. As can be seen in NMR spectra (Figure A3 and Figure A4) copolymers grafted on short polysiloxane chain (PMHS390): P3HT-Sil-PEG1, and P3HT-Sil-PEG2 contain very low amount of PEG chains. That is probably due to the tendency of P3HT chains to stack and be grafted next to each other on the PMHS backbone. Moreover, chloroform fraction chains of P3HT (longer and more regioregular than hexane fraction chains) should exhibit greater tendency towards π-stacking and agglomeration than hexane fraction chains, resulting in higher P3HT grafting density on PMHS and higher P3HT to PEG ratio in P3HT-Sil-PEG2 (bearing chloroform fraction P3HT chains) than in P3HT-Sil-PEG1 (bearing hexane fraction P3HT chains) (Table 1). On the other hand, using longer polysiloxane chain (PMHS1700—3200) as a backbone allows for obtaining copolymer with higher PEG content (Figure A5, Table 1).

**Figure 1:**
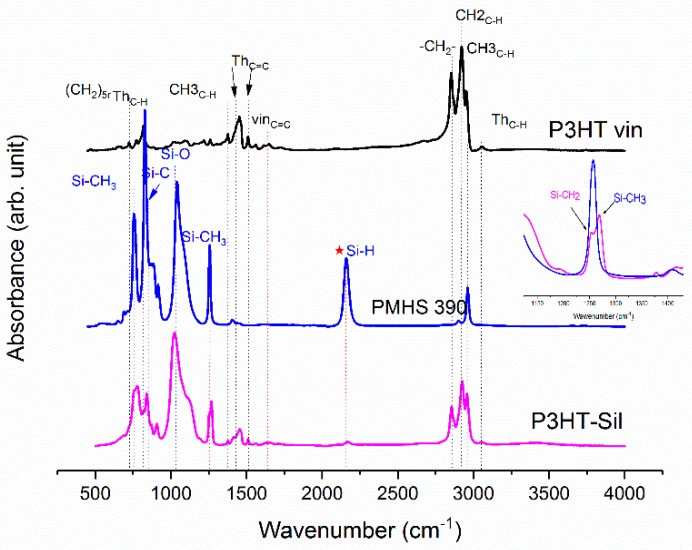
IR-ATR spectra of graft copolymer P3HT-Sil, vinyl terminated regioregular P3HT, and PMHS.

On Figure 1, the ATR-IR spectra of the P3HTvin, PHMS390, and the graft copolymer P3HT-Sil are presented.

The IR spectra of other copolymers are included in Appendix A. In the case of P3HT, the hexyl side chains give rise to the three bands at 3000–2850, 1378 and 728 cm^−1^. The peak at 1378 cm^−1^ is assigned to the deformation vibration of terminal methyl groups –CH_3_, while the peak at 728 cm^−1^ confirms the rocking vibration of hexyl substituent methylene groups –(CH_2_)_5_–. The peaks within the band of 3000–2850 cm^−1^ are typical for C–H bonds on the aliphatic side chain, which have been assigned respectively to the asymmetric C–H stretching vibrations of –CH_3_ (2953 cm^−1^) and –CH2– (2922 cm^−1^) moieties, as well as the symmetric C–H stretching vibration in –CH_2_– (2851 cm^−1^) moieties. The signal at 3057 cm^−1^ is assigned to the aromatic C–H stretching vibration of the thiophene ring, while the expected C–H out-of-plane vibration of a 2,3,5-trisubstituted ring is found at 828 cm^−1^. The absorption peak at 1460 cm^−1^ is associated with a symmetric C=C ring stretching vibration, while the peak at 1507 cm^−1^ is related to an asymmetric C=C ring stretching vibration. The presence of a vinyl end group is confirmed by the occurrence of C=C stretching vibration, situated at 1641cm^−1^.

The siloxane chains are confirmed by two broad bands at 1090 and at 1020 cm^−1^, which have been assigned to the Si–O–Si stretching vibrations in long chain siloxanes. The peaks at 800 and 900 cm^−1^ are related to Si–CH_3_ rock and Si–C stretch interactions. The signal at 1263 cm^−1^ is assigned to Si–CH_3_ symmetric stretching vibration in the methyl groups. The Si–H stretching vibration of PMHS is located at 2165 cm^−1^.

**Figure 2:**
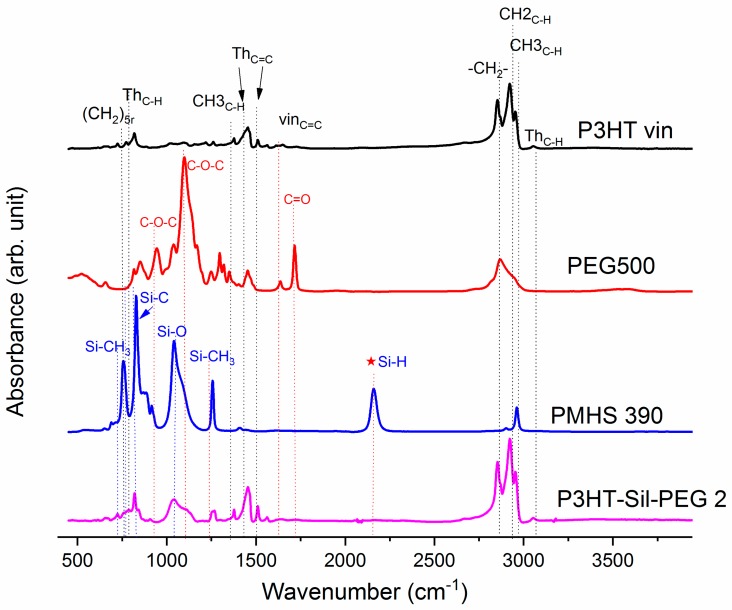
IR-ATR poly(methylsiloxane)-graft-poly(3-hexylthiophene)-graft-poly(ethylene glycol) (hexane fraction of vinyl terminated poly(3-hexylthiophene) P3HT-Sil-PEG2; poly(methylhydrosiloxane) trimethylsilyl terminated PHMS 390; poly(ethylene glycol) methyl ether methacrylate PEG 500.

The spectrum of PEG (Figure 2) shows the characteristic vibration bands of the CH_2_ and CH_3_ framework, stretching at 2883.5 cm^−1^, bending CH2 and CH3 at 1467.8 cm^−1^, and at 1342.4 cm^−1^, respectively, as well as C–O–C asymmetric and symmetric stretching at 1103.3 cm^-1^ and at 960.5 cm^−1^, respectively. The presence of a vinyl end group is also evidenced by the C=C stretching vibration found at 1641 cm^−1^.

The Si–H peak found for PHMS at 2160 cm^−1^ was reduced in the IR spectrum of P3HT-Sil and the signal of the vinyl groups at 1641 cm^−1^ from P3HTvin completely disappeared after hydrosilylation. Moreover, signals of Si–CH_2_–R wagging vibration at 1252 cm^−1^ appeared, as shown in Figure 1.

**Figure A6:**
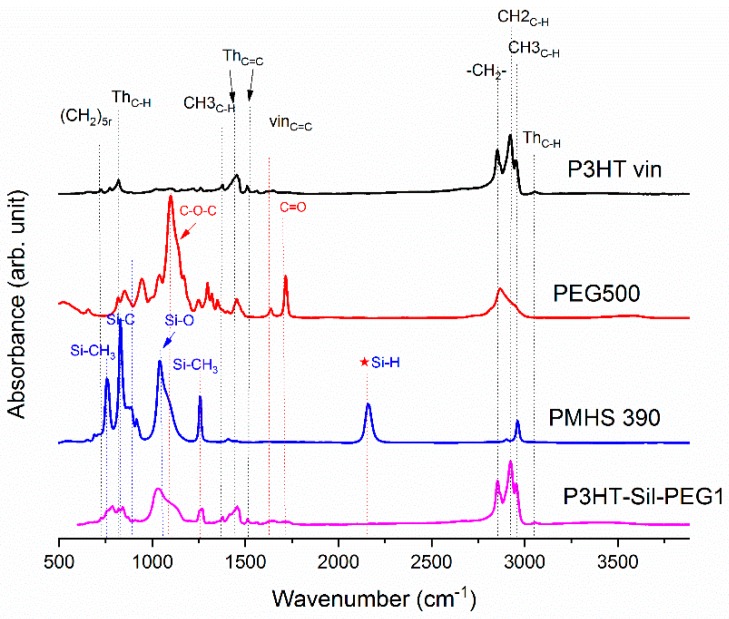
IR-ATR spectrum of poly(methylsiloxane)-graft-[poly(3-hexylthiophene; poly(ethylene glycol)] **P3HT-Sil-PEG1**;(hexane fraction of vinyl terminated poly(3-hexylthiophene); poly(methylhydrosiloxane) trimethylsilyl terminated PHMS 390; poly(ethylene glycol) methyl ether methacrylate PEG 500.

**Figure A7:**
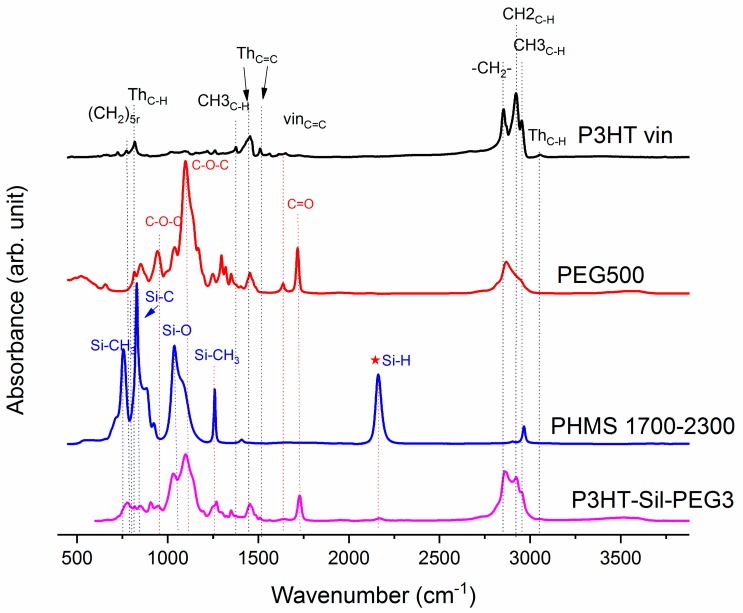
IR-ATR spectrum of poly(methylsiloxane)-graft-[poly(3-hexylthiophene); poly(ethylene glycol)] (chloroform fraction of vinyl terminated poly(3-hexylthiophene) (**P3HT-Sil-PEG3**); poly(methylhydrosiloxane) trimethylsilyl terminated PMHMS 1700–2300; poly(ethylene glycol) methyl ether methacrylate PEG 500.

The spectra of grafted copolymers not only displays all of the characteristic peaks of siloxane groups Si–O–Si at 1090 and at 1020 cm^−1^, as well as those of P3HT, but also shows the absorption bands of poly(ethylene glycol), such as CH_2_ and CH_3_ bending at 1467.8 cm^−1^ and at 1342.4 cm^−1^, C–O–C (see Figure A6 and Figure A7 in Appendix A). This indicates that P3HT and poly(ethylene glycol) were successfully grafted onto the polysiloxane chain.

### 3.2. AFM Microscopy

**Figure 3:**
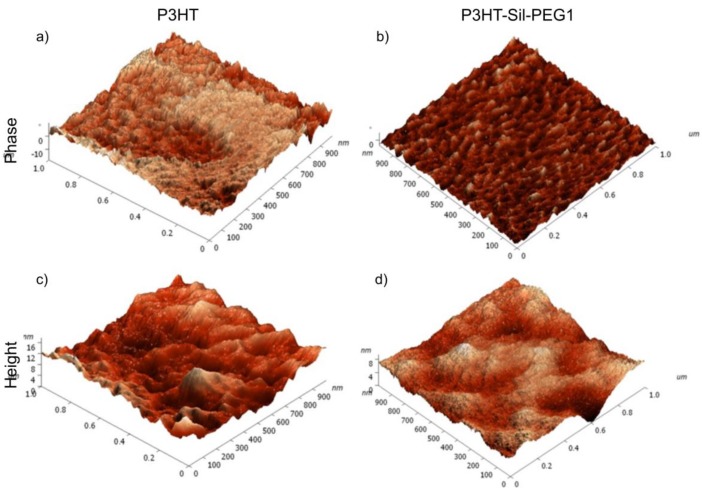
AFM phase: (**a**,**b**) and height: (**c**,**d**) images of P3HTvin: (**a**,**c**) and P3HT-Sil-PEG1: (**b**,**d**) on ITO electrodes. Images are of 1 μm × 1 μm regions.

Figure 3 shows the typical height and phase images of **P3HTvin** and **P3HT-Sil-PEG1** (image size 1 μm × 1 μm). From the phase images, highly ordered crystalline domains of **P3HTvin** are clearly visible in Figure 3a, but they are absent in Figure 3b. From the height images, the surface of **P3HTvin** is significantly rougher than that of **P3HT-Sil-PEG1**, with root-mean-squared surface roughness of 3.32 nm (Figure 3c) compared to 0.75 nm (Figure 3d). Islands and valleys are apparent in Figure 3c. These observations suggest that highly ordered **P3HTvin** chain alignment separated after molecular dilution of the conjugated system through grafting. It should be noted that grafted polymer films exhibited better flexibility and adhesion to the ITO/quartz electrode surface than **P3HTvin**.

### 3.3. Cyclic Voltammetry

**Figure 4:**
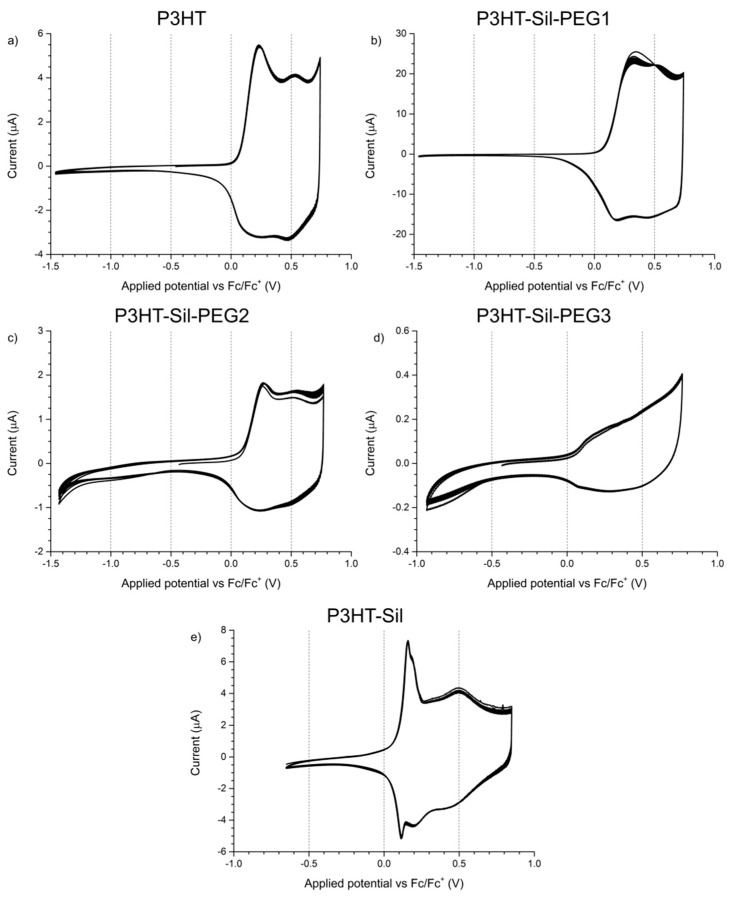
Electrochemical response of thin solid films of (**a**) regioregular P3HT; (**b**) P3HT-Sil-PEG1; (**c**) P3HT-Sil-PEG2; (**d**) P3HT-Sil-PEG3; (**e**) P3HT-Sil in 0.1M tetrabutylammonium hexafluorophosphate/acetonitrile. Potential scanning rate *r* = 0.1 V/s.

**Figure 5:**
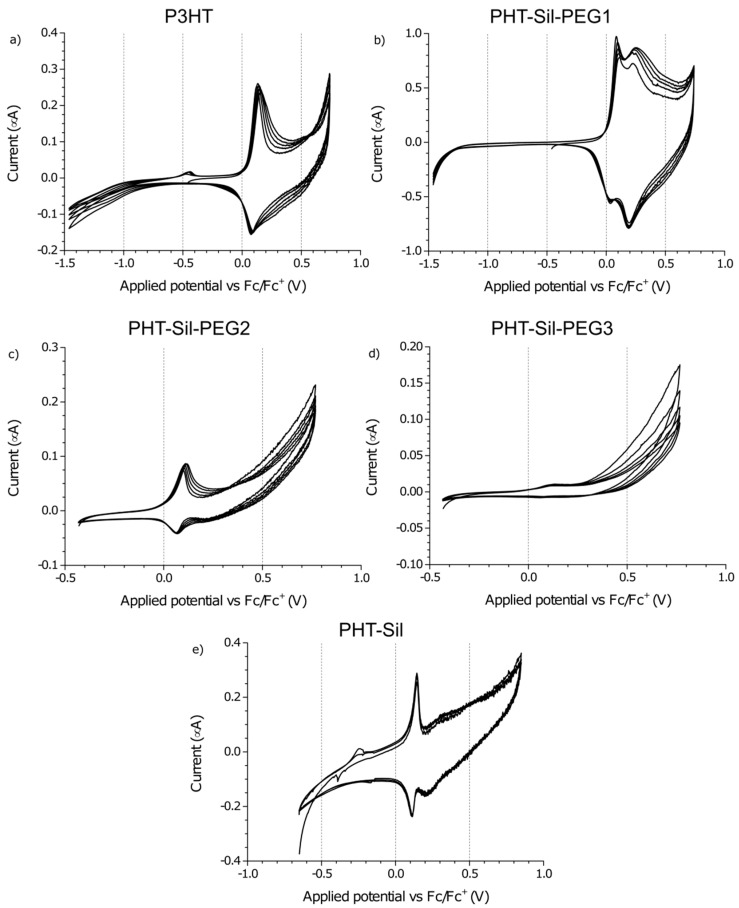
Electrochemical response of thin solid films of (**a**) regioregular P3HT; (**b**) P3HT-Sil-PEG1; (**c**) P3HT-Sil-PEG2; (**d**) P3HT-Sil-PEG3; (**e**) P3HT-Sil in 0.1M tetrabutylammonium hexafluorophosphate/acetonitrile. Potential scanning rate *r* = 0.005 V/s.

The electrochemical response of **P3HTvin** films on a platinum electrode was observed at potential scanning rates of 0.1 V/s (Figure 4a) and 0.005 V/s (Figure 5a). In the case of the experiment using a scanning rate of 0.1 V/s, two distinct redox systems are found, with their oxidative peaks being observed at +0.24 V and at +0.53 V, as well as a slope with an onset at approximately +0.65 V, similar to what is reported in literature for P3HT and other 3-alkylthiophene polymers [9,20,21,22,23]. The two redox systems are typically attributed to transitions between ground state polymer segments and polaron- and bipolaron-bearing segments. Interestingly, it can be shown by simulation that, in the case of the low potential redox pair, both the oxidation and reduction signals are composite, each consisting of two individual signals, roughly 0.09 V apart. The composite nature can originate from the existence of crystalline and amorphous regions in the polymer film, exhibiting different alkyl chain packing and thiophene ring stacking schemes, affecting their electronic properties [24,25,26,27,28,29,30].

The oxidative slope, in turn, is commonly associated with polymer over-oxidation, as extending the applied potential range towards more positive potentials results in a rapid loss of the electrochemical response of the polymer in subsequent potential cycles, similar to what was found for polythiophene [31]. The occurrence of over-oxidative deactivation is effectively the upper limit of the operating potential window for conjugated polymer materials, as this process is irreversible. Consequently, the potentials applied to the investigated polymer films were adequately limited (to, at most, approximately +0.7 V vs. Fc/Fc+, based on the abovementioned report), so as to minimize, if not eliminate, the influence of such over-oxidation phenomena. The overall shape of the CV curve—narrow at low potentials and broadly rectangular at more positive potentials—indicates that a significant change in the conductance of the system takes place during the measurement, which is the expected consequence of doping/dedoping the conjugated polymer film.

When the electrochemical response of **P3HTvin** is observed at a 0.005 V/s scan rate, the features of the CV curve (Figure 5a) are a single oxidation peak at +0.13 V and a single reduction peak at +0.08 V. The over-oxidative slope is present and appears to be more pronounced due to the absence of the second pair of redox signals at higher potentials. Interestingly, in subsequent potential cycles, the shape of the response is largely maintained, with the redox pair signals broadening slightly towards the high potential end of the voltammogram. After multiple potential cycles, an inflection point evolves at approximately +0.58 V, likely indicating an obscured oxidation signal. Both the broadening and the appearance of the inflection point at +0.58 V imply that the polymer undergoes reorganisation during repeated doping/dedoping, altering either the feasibility or the kinetics of the second doping stage. Similar trends are seen in the other investigated polymers upon repeated potential cycling, but the new peak is formed only in the case of **P3HTvin**.

The shape of the CV curves recorded for **P3HT-Sil-PEG1** films (Figure 4b) differs from that of regioregular P3HT films. In the first potential cycle, a single broad peak is initially observed at +0.34 V, evolving into two distinct peaks at +0.33 and +0.53 V in subsequent cycles, indicating that reorganisation of the graft copolymer takes place upon doping/dedoping. Although the low potential peak is located at significantly higher potentials than the corresponding signal for **P3HTvin** (+0.24 V), making the material initially more resistant to oxidation, the high potential peak is found at +0.53 V, virtually identical to the corresponding signal for **P3HTvin**. Interestingly, while the ground states of the two polymers differ significantly, their p-doping appears to give rise to electronically similar doped states. Similarly to **P3HTvin**, the low-potential end of the voltammogram is relatively narrow, while the high-potential end is significantly wider. Although it is only a very rough measure of changes in the conductance of a polymer film, this effect is much more pronounced in the case of **P3HT-Sil-PEG1**, with the CV width at high potentials being almost two hundred times the width at low potentials, suggesting a more significant change in relative conductance of the polymer upon doping than for **P3HTvin**.

The CV curve recorded at the 0.005 V/s scan rate (Figure 5b), unlike that of **P3HTvin**, features an additional pair of sharp redox signals, with the oxidation peak being observed at +0.10 V. This phenomenon was encountered earlier, by Baranowska and Łapkowski [32] in their studies on 2,2′-diquinoxalyl and was ascribed to changes in the adsorption of the molecule on the working electrode. Similarly, for **P3HT-Sil-PEG1**, these sharp CV peaks can be attributed to changes in the conformation of the grafted P3HT chains. This type of conformation changes is, however, unlikely for well-ordered P3HT chains, due to stabilising interactions between nearby P3HT chains. Consequently, the changes in conformation would be expected to arise from segments of the copolymer bearing isolated P3HT chains, due to non-uniform grafting.

Due to the high share of 3HT units in the structure of **P3HT-Sil-PEG2**, its electrochemical response (Figure 4c) is more similar to that of **P3HTvin** than to that of **P3HT-Sil-PEG1**. In the oxidation half-cycle, two oxidation peaks, at +0.26 and +0.52 V respectively, and a high potential over-oxidative slope are found, whereas the reduction half-cycle features a single reduction peak at +0.23 V and an inflection point, indicative of an overlapping higher potential reduction process, at +0.46 V. The low potential oxidative peak is found at a slightly higher potential than in the case of **P3HTvin** or **P3HT-Sil-PEG1**, indicating that a slightly higher energetic barrier needs to be overcome to introduce charges onto the grafted macromolecule, due to the lessened stabilising effect of interactions between P3HT chains. Nevertheless, the high potential oxidative peak is found at a potential similar to that of the aforementioned polymers. This, in turn, implies that a p-doped state, similar to that of **P3HTvin** is achieved for both **P3HT-Sil-PEG1** and **P3HT-Sil-PEG2**.

The 0.005 V/s CV of P3HT-Sil-PEG2 (Figure 5c) is relatively similar to that of the **P3HTvin** film. Interestingly, the magnitude of the peak currents, in relation to the currents of the oxidative slope is noticeably lower than in the case of P3HT.

In the case of **P3HT-Sil-PEG3** (Figure 4d), the low P3HT content in the copolymer gives rise to a weak electrochemical response, showing only poorly developed features. Even so, the observed broad oxidative signal centred at +0.19 V and an inflection point at +0.55 V, hint at the occurrence of doping/dedoping processes, with the potentials being similar to those observed for **P3HTvin** and other **P3HT-Sil-PEG** copolymers.

Investigating the response of the P3HT-Sil-PEG3 film at the lower potential scanning rate (Figure 5d) reveals that by lowering the P3HT grafting density, it is possible to significantly change the properties of the copolymer. The CV features an oxidation signal at +0.13 V and only a trace of a reduction signal at +0.06 V. This is consistent with the abovementioned lowering of peak current in relation to the magnitude of the oxidative slope currents, observed for P3HT-Sil-PEG2.

The electrochemical response of **P3HT-Sil** (Figure 4e) is particularly interesting among the investigated systems, as not only does it show the redox signals expected for a P3HT derivative, but an additional pair of sharp signals, centred at approximately +0.14 V, is also found, as in the case of the 0.005 V/s CV of **P3HT-Sil-PEG1**. Assuming that the P3HT chains are indeed grafted non-uniformly, giving rise to segments of the copolymer bearing “interacting” and isolated P3HT chains, this result is within expectations. Interestingly, the fact that this feature is observed at different potential scanning rates for different copolymers is evidence that the presence or lack of co-grafts affects the kinetics of this conformational change. This difference in kinetics is most likely brought on by the additional PEG grafts, hindering changes in the conformation of neighbouring P3HT chains, both due to steric factors and to electrostatic interactions with the ether groups of PEG.

The 0.005 V/s response of **P3HT-Sil** consists of a pronounced redox pair (centred at approximately +0.13 V), as is the case for most other investigated copolymers, but also shows a faint oxidative peak at +0.32 V, coupled with a more pronounced reduction signal at +0.21 V. 

### 3.4. EPR-UV-Vis-NIR Spectroelectrochemistry

**Figure 6:**
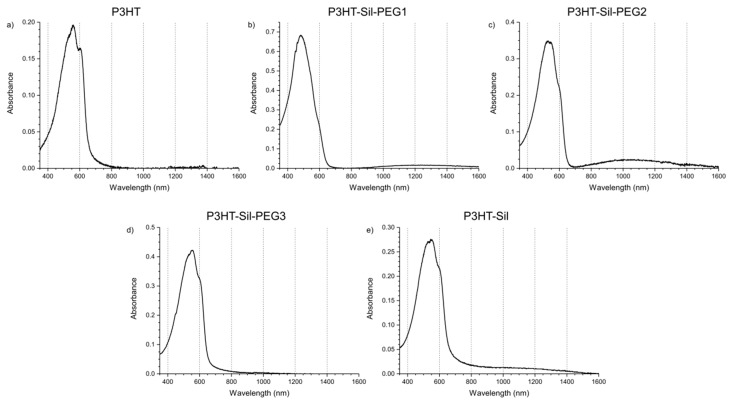
UV-Vis-NIR spectra (corrected for supporting electrolyte absorption) of investigated polymer films of (**a**) regioregular P3HT; (**b**) P3HT-Sil-PEG1; (**c**) P3HT-Sil-PEG2; (**d**) P3HT-Sil-PEG3; (**e**) P3HT-Sil in a solution of 0.1M tetrabutylammonium hexafluorophosphate in acetonitrile, at open circuit potential.

The UV-Vis-NIR spectrum (Figure 6a) of the non-polarised **P3HTvin** film on ITO/quartz consists of a broad absorption signal, with a slope at 655 nm, corresponding to an optical band gap of 1.89 eV. The absorption signal is composite, with three individual, overlapping absorption peaks being readily identified at 601, 561, and 535 nm, corresponding to transition energies of 2.06, 2.21, and 2.32 eV respectively. The slight differences in the absorption peak wavelengths and, therefore, energies (0.15 and 0.11 eV from the middle peak respectively) of the respective transitions likely arise due to vibronic splitting of the main transition. An interesting explanation for the origin of this signal shape has been offered by Ludwigs et al. [33], attributing the main transition signal to the response of amorphous segments of the polymer and the vibronic signals to the ordered segments. We have utilized ^1^H-NMR and IR spectroscopy to confirm this, finding no evidence that these signals arise either from impurities bearing a conjugated bond system or defects in the P3HT chains. The absorption signals are also unlikely to arise from chains of sufficiently varying length, due to both the rather high molecular weight of our **P3HTvin** and to the post-synthesis purification scheme involving fractional extraction, yielding samples with narrower molecular weight distributions than the raw synthesis product. Upon the application of positive potentials to the polymer films, these component signals decline at different rates.

The absorption spectra of the grafted copolymers were recorded immediately after deposition and for the films being immersed in a solution of the supporting electrolyte in open circuit conditions (Figure 6b–e). Vibronic splitting, observed for **P3HTvin** films (Figure 6a), can also be found for the graft copolymer films (Figure 6b–e), manifesting most noticeably for the **P3HT-Sil-PEG3** and **P3HT-Sil** films, with the other two films exhibiting slightly lesser differences in the energy of the individual absorption signals, possibly hinting at a difference in the degree of freedom of rotational and translational movement of the P3HT chains. 

**Figure 7:**
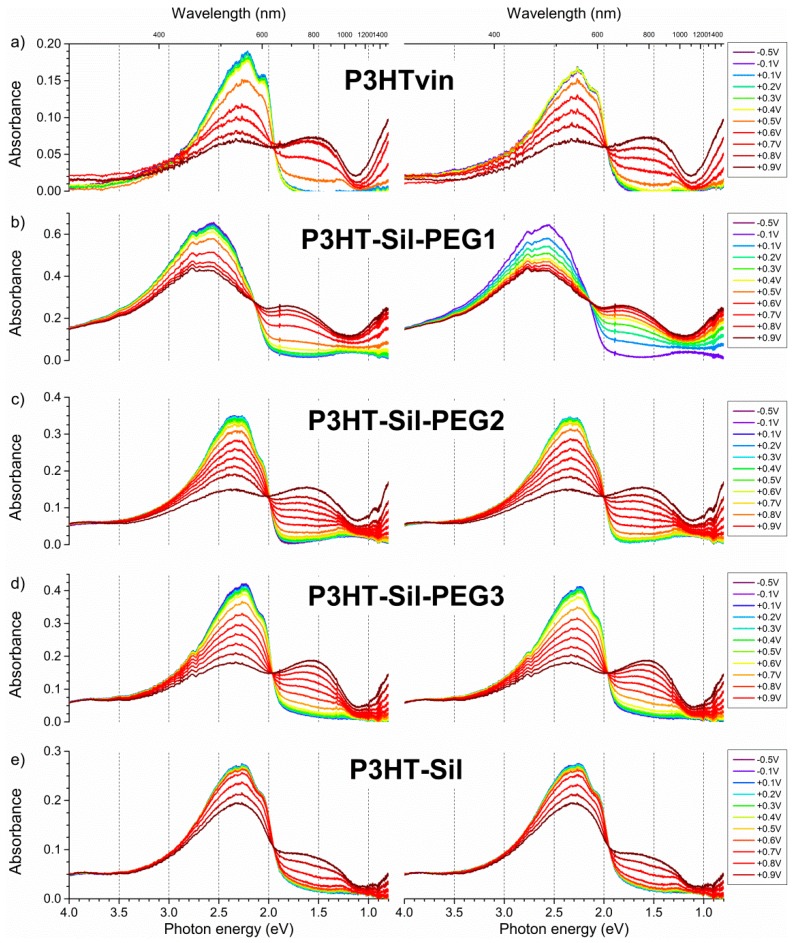
UV-Vis-NIR spectra recorded during the application of stepwise increasing (**left**) and decreasing (**right**) potential (vs. Ag/Ag+) to films of the investigated polymer and copolymer: (**a**) regioregular P3HT; (**b**) P3HT-Sil-PEG1; (**c**) P3HT-Sil-PEG2; (**d**) P3HT-Sil-PEG3; (**e**) P3HT-Sil deposited on ITO/quartz electrodes.

**Figure 8:**
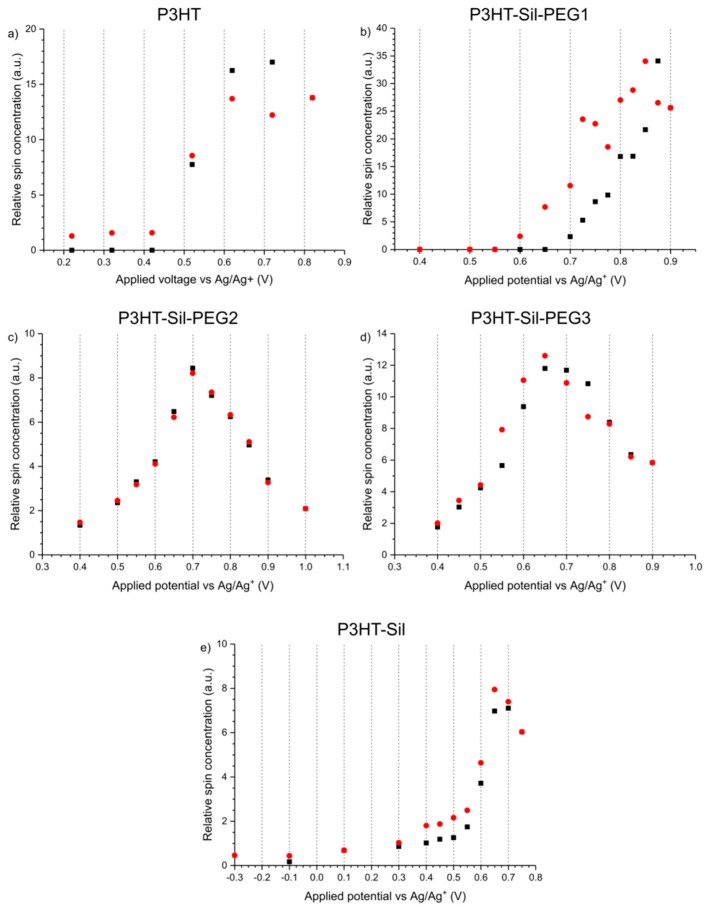
Stepwise EPR spectroelectrochemistry of (**a**) regioregular P3HT; (**b**) P3HT-Sil-PEG1; (**c**) P3HT-Sil-PEG2; (**d**) P3HT-Sil-PEG3; (**e**) P3HT-Sil films on a Pt electrode. Black points denote increasing applied potential (p-doping), whereas red points denote decreasing applied potential (dedoping).

When increasingly positive potentials are applied to a **P3HTvin** film (Figure 7a, left), the intensity of the three signals, constituting the absorption spectrum, decreases non-uniformly. The component signal at 601 nm appears to deteriorate most rapidly, resulting in a blue shift of the composite signal absorption maximum. When +0.5 V is applied to the polymer film, a broad absorption band evolves at 800 nm, being accompanied by a sharp increase in the observed relative spin concentration (Figure 8a). The presence of this band along with that of a discernible component signal at 950 nm, rather than a single absorption peak, can imply that different doped state structures are at least transitionally present. The band and additional signal develop into a single apparent peak when +0.7V is applied to the film. Incidentally, a maximum spin concentration is predicted at approximately this potential, indicating the highest concentration of polaronic charge carriers in the polymer film. Upon reducing the applied potential (Figure 7a, right), the observed spectral changes are partially reversed as the polymer is dedoped, however, the intensity of the 950 nm signal is noticeably lower, further supporting the claim of the occurrence of conformational changes in the polymer film at higher doping levels. Furthermore, not only do trace absorption signals remain in the NIR range even at potentials as low as −0.5 V, but also the absorption signal of the undoped polymer is not fully restored. Similarly, a detectable concentration of spin-bearing species remains in the polymer film (Figure 8a). Although these features could imply decay of the polymer film and formation of spin-bearing degradation products, incomplete dedoping of the polymer film is more likely, as the absorption signals of these species occur in the NIR range, indicating a relatively long conjugated bond system, rather than in the UV-Vis range, as would be expected for degradation products with shorter conjugation lengths.

Upon application of increasingly positive potentials, **P3HT-Sil-PEG1** films show a small red shift of the undoped polymer signal, due to the non-uniform decay of the individual absorption signals, similar to that seen for **P3HTvin**, but not as pronounced. Similar to what was seen for **P3HTvin**, changes in the spectrum of the polymer film start manifesting visibly at above + 0.6 V, with a broad shoulder developing in the range of 600–800 nm, which evolves into a broad peak when more positive potentials are applied. These spectral changes are accompanied by an increase in the relative spin concentration observed in the coupled EPR experiments (Figure 8b), evidencing the electrochemical generation of spin-bearing polaronic species. Simultaneously, absorption signals begin developing in the NIR range, with a projected signal maximum at approximately 2100 nm, well beyond the spectral range of our experimental setup. These signals can be attributed to bipolaronic charge carriers, indicating that the two types of charged species are being generated alongside each other. Interestingly, both absorption signals continue to gain intensity, even in the final potential steps to +0.9 V, which brings about a sharp decrease in spin concentration, and to +1.0 V, indicative of the transition from polarons to bipolarons as the primary charge carriers in the polymer.

When the direction of potential staircasing is reversed and progressively less positive potentials are being applied, the abovementioned absorption bands, attributed to polarons and bipolarons, begin declining as the initial shape of the absorption spectrum is restored. Although the dependence of relative spin concentration on applied potential evidences a minor doping level hysteresis, below +0.5 V no spins can be detected, unlike **P3HTvin** and **P3HT-Sil**, which show persistent spin concentrations even at negative potentials (Figure 8e). This, along with the near perfect restoration of the original absorption spectrum after the experiment, shows that both over-oxidative deactivation of the polymer film and charge trapping have a much lesser influence on the doping/dedoping of **P3HT-Sil-PEG1** polymer films than on **P3HTvin** films. This is even more significant if the absorbances of the undoped polymer layers are compared—0.19 and 0.64 for **P3HTvin** and **P3HT-Sil-**PEG1 respectively (Figure 7). If we assume that the molar absorption coefficients for the P3HT chains are in both cases comparable, the thickness of the **P3HT-Sil-PEG1** layer would be expected to be roughly three times greater than the thickness of the **P3HTvin** film. This significant thickness would be expected to severely hinder doping/dedoping reversibility; even despite this, P3HT-Sil-PEG1 shows better doping reversibility than **P3HTvin**.

In the case of **P3HT-Sil-PEG2**, **P3HT-Sil-PEG3**, and **P3HT-Sil**, very similar results are obtained in terms of the UV-Vis-NIR spectra, but virtually no doping level hysteresis is observed. The dependence of relative spin concentration on applied potential (Figure 8c,d) for **P3HT-Sil-PEG2** and **P3HT-Sil-PEG3** layers is such that the data points observed when the applied potential is staircased towards positive potentials almost perfectly match the data points recorded when the potential is staircased back to lower potentials. In both cases, a spin concentration maximum is observed, respectively at +0.7 V and at +0.65 V for **P3HT-Sil-PEG2** and for **P3HT-Sil-PEG3**.

In the case **of P3HT-Sil**, the maximum applied potential was severely curtailed due to the fact that irreversible changes had begun occurring in the copolymer film at higher potentials; this is most likely due to the electrolysis products reacting with the active Si–H sites (or their derivatives produced by reaction with air and moisture) that were not substituted with P3HT chains and, unlike the **P3HT-Sil-PEG** class, were also not substituted with PEG chains. Interestingly, for **P3HT-Sil**, the dependence of spin concentration on applied potential is intermediate between that of **P3HTvin** and those of the **P3HT-Sil-PEG** class; as in the case of the latter, only marginal doping state hysteresis is observed, while, as is the case for **P3HTvin**, generated spins persist in the material even at strongly negative potentials. This implies that, while grafting P3HT chains on the polysiloxane scaffold improves doping/dedoping reversibility, the preventative influence of PEG chains on the formation dedoping-resistant charge carriers is also significant.

### 3.5. Time-Resolved UV-Vis Spectroelectrochemistry

**Figure A8:**
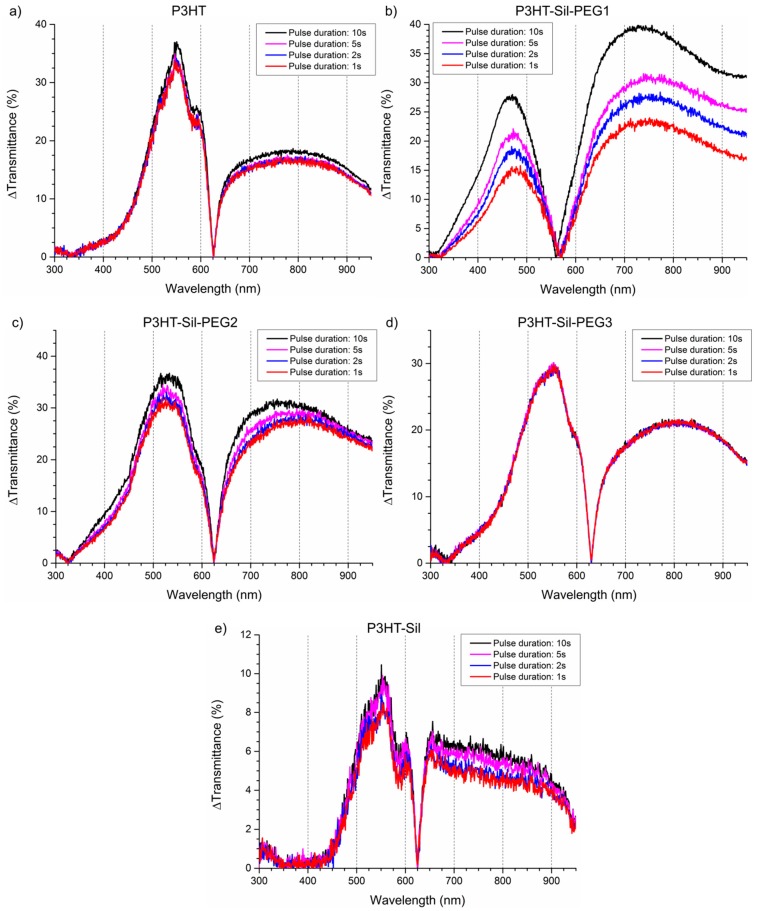
Colour contrasts (given as the absolute value of transmittance differences between the undoped and doped states) plotted versus wavelength for films of the investigated copolymers: (**a**) P3HT; (**b**) P3HT-Sil-PEG1; (**c**) P3HT-Sil-PEG2; (**d**) P3HT-Sil-PEG3; (**e**) P3HT-Sil.

Time-resolved UV-Vis spectroelectrochemistry was employed to investigate the electrochromic capabilities of the synthesised copolymers in relation to those of **P3HTvin**. The experiments utilised two types of electrochemical stimuli: the standard approach of alternating between two potential values with a fixed step width (i.e. multi-step chronoamperometry), and a slow sweep between the two boundary potentials (using cyclic voltammetry). The latter type of stimuli, with its slow potential changes, was introduced as means of approximating a “near-equilibrium” doping/dedoping process. The absorbance of the undoped polymer absorption signal maximum was used to compare the apparent thickness of the polymer film, based on the assumption that the molar absorption coefficient for the P3HT chains is within a single order of magnitude regardless of whether the chain is a free P3HT chain or one grafted to the polysiloxane scaffold. Although control over the thickness of the polymer films was acceptable, it was not precise enough to allow obtaining exactly the same absorbance values for all samples, even with a large number of films being deposited. As such, several polymer films were investigated for each macromolecule (Table 2), typically a “thin” and a “thick” film, with some more extreme samples being included in the comparison as well. The dependence of the achieved optical contrast on the wavelength is shown in detail in Figure A8 in Appendix A.

**Figure 9:**
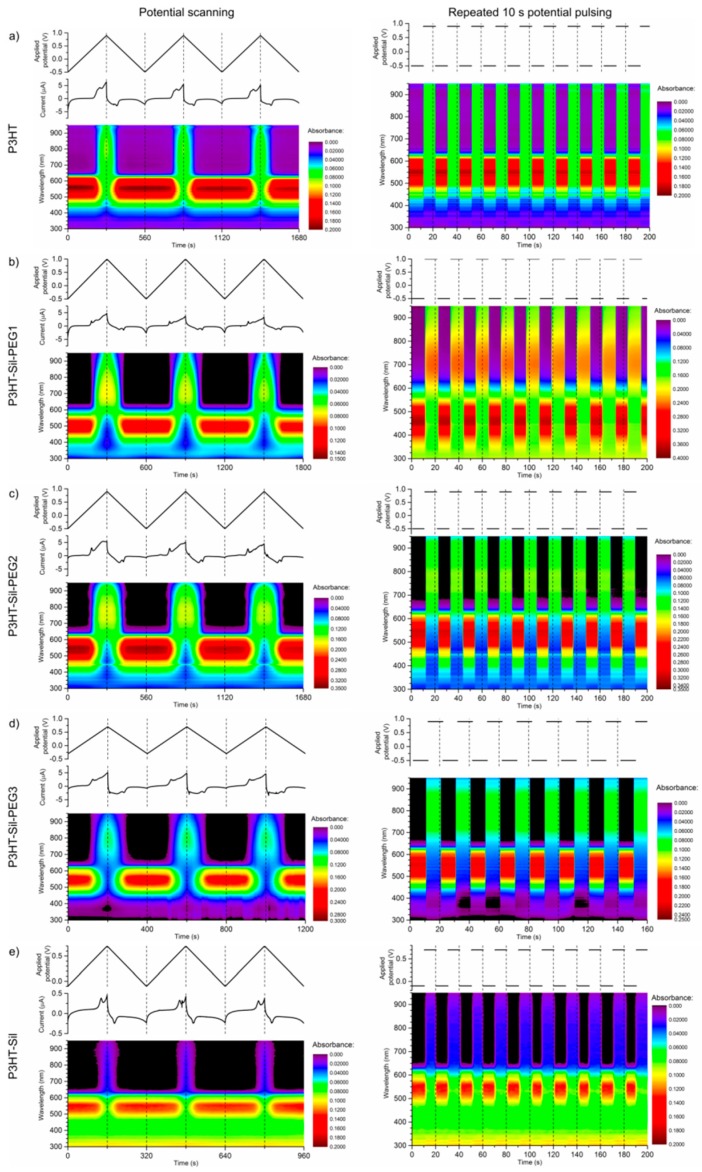
Time-resolved UV-Vis spectra recorded during: potential scanning at a rate of 0.005 V·s^−1^ (left) and repeated potential switching (right), with a 10s pulse duration of the investigated polymer and copolymer films: (**a**) regioregular P3HT; (**b**) P3HT-Sil-PEG1; (**c**) P3HT-Sil-PEG2; (**d**) P3HT-Sil-PEG3; (**e**) P3HT-Sil

Relatively high optical contrasts are observed for the polymer films, with the **P3HT-Sil-PEG** class compounds generally outperforming **P3HTvin** films (Figure 9) of a similar apparent thickness. The **P3HT-Sil** system, lacking PEG grafts, in turn shows slightly worse performance, although the experiments were conducted in a narrower range of potentials than in the case of the other polymer films. Switching times for **P3HTvin** and **P3HT-Sil-PEG3** are below 1 s (assuming a criterion that at least 90 % of the contrast, observed in the 10 s pulse width switching experiment, is achieved), whereas a switching time in the range of 2–3 s is seen for **P3HT-Sil-PEG2** and **P3HT-Sil**. In the case of **P3HT-Sil-PEG1**, despite it achieving the highest contrast values, the switching time is in excess of 5 s.

### 3.6. Conductance of Polymer Films

**Figure 10:**
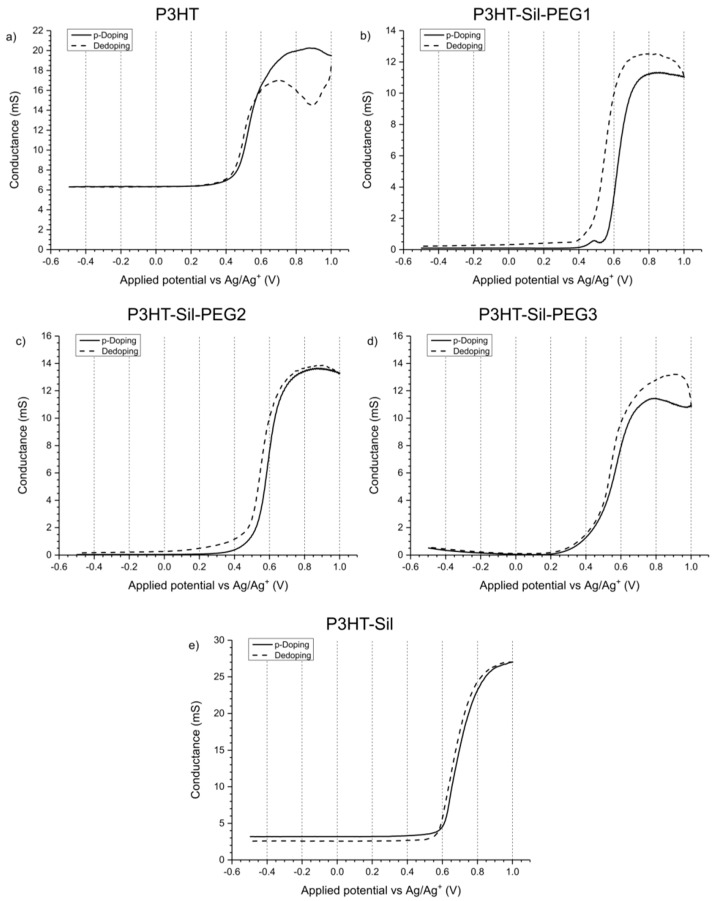
Double working electrode electroconductometry of (**a**) regioregular P3HT; (**b**) P3HT-Sil-PEG1; (**c**) P3HT-Sil-PEG2; (**d**) P3HT-Sil-PEG3; (**e**) P3HT-Sil films on an interdigitated Pt electrode. Potential offset between the working electrodes Δ*V* = 0.005 V.

The conductance of **P3HTvin** (Figure 10a) is, interestingly, noticeable even at low potentials, indicating the existence of stable charge carriers, concurrent with our expectations. Upon application of increasingly positive potentials to the working electrodes, conductance of the polymer film increases sharply, corresponding to the electro-generation of numerous polaronic and bipolaronic charge carriers, with polarons being expected to be dominant charge carriers at low doping levels. At higher doping levels, however, the conductance of the polymer starts to slowly decline, even though these should correspond to the formation of bipolaronic charge carriers. A similar phenomenon was encountered for polythiophene by Harima et al. [34], who ascribed it to over-oxidation of the polymer film. It is worth noting that the curves showing the dependence of conductance and apparent positive charge carrier mobility on doping level had nearly identical shape. As such, the source of this high-potential conductance decline is a decline in average charge carrier mobility rather than a drop in the overall population of charge carriers, which would be expected if over-oxidative deactivation of the polymer was significant at these doping levels. 

The observed decline in conductivity is, therefore, likely due to the difference in mobility between bipolaronic and polaronic charge carriers. This explanation is again brought to mind, when we reverse the potential scan polarity—initially we see a sharp drop in conductance due to the decay of bipolarons. When lower potentials are applied, however, an increase in conductance is observed, peaking at +0.7 V. This peak in conductance coincides with a maximum concentration of spin-bearing species observed via EPR spectroelectrochemical measurements (Figure 8a). By applying potentials below +0.7 V we dedope the polymer, with both conductance and spin concentration steadily decreasing.

Interestingly, when lower potentials, in the range of 0.2 to −0.5 V are applied, the conductance of the polymer film begins declining only marginally, indicative of an on-going charge de-trapping process. A low amount of spin centres also persists at these potentials, registered by EPR spectroelectrochemistry. The observed residual conductance, indicates that complete dedoping of the oxidised polymer segments does not take place, which could be interpreted as a symptom of charge trapping [35,36,37]. For a typical conjugated polymer, at such negative potentials, we would expect to see a gradual decline in conductance of the polymer film (due to reduction of positive charge carriers in the polymer film) rather than the almost unchanging and relatively high observed conductance value. This value is by far too high to be accounted for solely by the charge carriers generated in situ due to reactions with oxygen. This implies that the “residual” charge carriers are tied to polymer segments, whose oxidised form is incapable of being reduced at the employed range of potentials.

In the case of **P3HT-Sil-PEG** copolymers (Figure 10b–d), the conductance of the undoped polymer film is initially much lower (on the order of 0.05–1 mS) than that of **P3HTvin**. This can be attributed both to a lesser amount of charge carriers in the polymer film and to the effect of the polysiloxane segments present in the structure of the copolymers, acting as partial insulation between the electrode and conjugated polymer segments. In light of these observations, this copolymer would appear be much more resistant to reactions with oxygen than **P3HTvin**, seeing that it shows no significant conductance in the “undoped” state. Upon application of increasingly positive potentials to the working electrodes, the conductance either decreases slightly (**P3HT-Sil-PEG1** and **P3HT-Sil-PEG3**) or is maintained (**P3HT-Sil-PEG2**). This is likely dependent on the population of charge carriers in the untreated copolymer films, with the initially strongly negative potentials being able to remove some of those charge carriers. Once potentials above +0.2 V (+0.4 V in the case of **P3HT-Sil-PEG1**) are applied, conductance begins sharply increasing, as positive charges are being injected onto the polymer chains. At approximately +0.6 V, the conductance of all the copolymers stabilises and changes only marginally when more positive potentials are being applied. Despite this stabilisation of conductance values, the UV-Vis-NIR absorption of doped copolymer segments keeps increasing for all three **P3HT-Sil-PEG** copolymers, implying that new charge carriers are being introduced. This indicates that the apparent mobility of charge carriers must be declining, for conductance to remain at roughly stable values.

Upon reversing the potential scan polarity, we observe a further growth of conductance of the polymers, rather than the expected decline due to dedoping. Interestingly, conductance is higher across all applied potentials and, only in the case of **P3HT-Sil-PEG1**, coincides with noticeably higher spin concentrations than what has been observed in the doping half-cycle (Figure 8b). As such, we cannot ascribe the increased conductance only to a difference in spin-bearing/spinless charge carrier populations. Among the results of the spectroelectrochemical investigations of these copolymer films, two primary features can be used to explain this conductance increase, also for the two other copolymers:

- Cyclic voltammetry curves recorded at low potential scanning rates (Figure 8) show a difference in the magnitudes and shapes of the oxidation and reduction peaks of **P3HT-Sil-PEG2** and **P3HT-Sil-PEG3** films, whereas for the **P3HT-Sil-PEG1** films, a sharp redox pair is seen, which is ascribed to a change in the conformation of isolated **P3HTvin** grafts upon doping;
- No noticeable discrepancies are observed between the UV-Vis-NIR spectra (Figure 7) of **P3HT-Sil-PEG2** and **P3HT-Sil-PEG3** films recorded prior to and following the application of electrochemical stimuli.

The abovementioned features point to the restructuring of the different layers constituting the films, which results in the formation of conductive paths in its bulk, rather than reorganisation of the structure of individual macromolecules. This restructuring is consistent with the changes of the CV curve shape, observed when the polymer films are subjected to potential cycling at a rate of 0.005 V/s (Figure 5). As such, this restructuring may be tied to the conformation of the non-conjugated polysiloxane and poly(ethylene glycol) chains, which are suspended in a P3HT-rich environment, whose polarity changes significantly upon doping, inducing changes in their structure.

Interestingly, in the case of **P3HT-Sil** (Figure 10e), which lacks the non-conjugated chains, we initially observe a non-zero conductance value, almost as high as for **P3HTvin**, followed by a sharp increase when higher potentials are applied. As such, it would appear that the resistance of the copolymer to reactions with oxygen is largely bestowed by the non-conjugated PEG chains. When the maximum applied potential is achieved and we begin decreasing it, the polymer film initially exhibits higher conductance than in the doping half-cycle, much like for the **P3HT-Sil-PEG** copolymers. At lower potentials, the conductance drops below the initial value, indicating that some of the charge carriers present in the polymer film were “detrapped”, which may also be a result of doping-induced reorganisation of the spatial structure of the film.

The doped/undoped (“ON/OFF”) conductance ratio (Table 3) for the grafted **P3HT-Sil** is higher than for **P3HTvin** itself, but still is sharply lower than the ratio for the PEG-bearing copolymers, evidencing their great change in conductance upon doping/dedoping. Interestingly, the highest ratios were observed for **P3HT-Sil-PEG2** and **P3HT-Sil-PEG3**, the polymers showing the most reversible behaviour (particularly so in terms of their spin concentrations, Figure 8).

By hindering the inter-chain π-π interactions and introducing polyelectrolyte-capable PEG moieties we increase the resistance of the polymers to irreversible reactions with oxygen and facilitate the removal of charge carriers during dedoping. This indicates that the HOMO levels of the copolymers are at a higher energy than HOMO of P3HT, since the copolymers are less susceptible to reactions with oxygen.

### 3.7. Solar Cells

**Figure 11:**
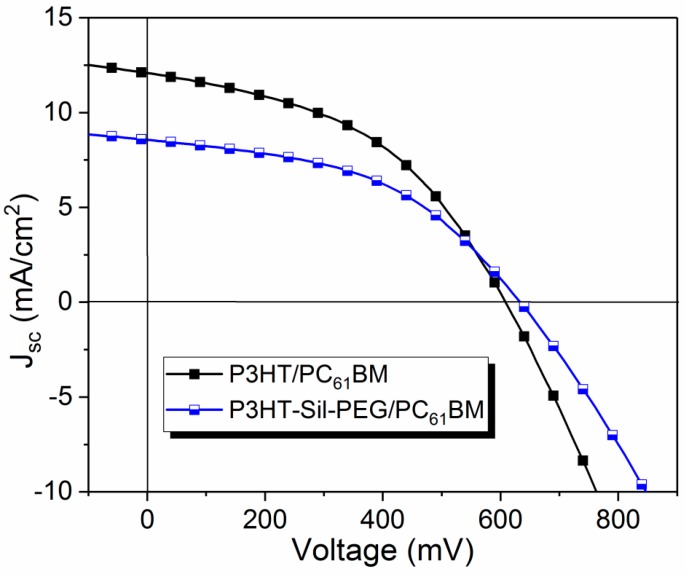
Current density–voltage (J–V) curves of device based on P3HT-Sil-PEG3:PC_61_BM BHJ and reference P3HT:PC_61_BM BHJ.

The devices with the structure ITO/PEDOT:PSS/P3HT-Sil-PEG3:PC_61_BM/Al, in which **P3HT-Sil-PEG3** served as the electron donor and PC_61_BM as the electron acceptor were fabricated, in order to investigate the possible usefulness of the studied polymers as active materials in BHJ solar cells. Devices were fabricated without optimization. Devices containing PC_61_BM and P3HT were chosen as reference. In both cases donor material/PC_61_BM 2:1 weight ratio was chosen. Current density–voltage (J–V) curves are shown in Figure 11.

The basic photovoltaic parameters are summarized in Table 4. Power conversion efficiency (PCE) of 2.46 % was achieved for the best device containing **P3HT-Sil-PEG3**. This device shows a Fill Factor (FF) of 0.46, open circuit voltage (*V*_oc_) of 0.63 V. Similar results were obtained for the reference devices with FF of 0.45 and *V*_oc_ of 0.62 V. The difference was in short circuit current (*J*_sc_) with 8.57 mA/cm^2^ for **P3HT-Sil-PEG3** based BHJ comparing to reference device with *J*_sc_ of 12.58 mA/cm^2^.

Although the devices with a **P3HT-Sil-PEG3**/PC_61_BM layer present lower efficiency than the reference devices, based on P3HT/PC_61_BM BHJ, the results are very promising because the **P3HT-Sil-PEG3**/PC_61_BM BHJ contains significantly less P3HT than the P3HT/PC_61_BM reference. Compared to other works on conductive copolymers (Table 4), similar photovoltaic results were obtained. Moreover, further optimisation of devices and polymer structure can lead to improvement of the performance of the device. This indicates great potential of the studied polymers for their application in photovoltaics.

## 4. Conclusions

Summarising, we have demonstrated a synthetic pathway to a new class of copolymers, realising molecular dilution of the conjugated system through grafting onto a polysiloxane scaffold alongside non-conjugated polymer chains. The synthesised **P3HT-Sil-PEG** and **P3HT-Sil** systems retain the electroactivity and stability of their parent system—regioregular poly(3-hexylthiophene). 

Regioregular P3HT is known to form lamellae [41] due to the π–π interactions between the individual chains. Through numerous such interactions, charge generated on one polymer chain may be delocalised not only over its neighbouring repeat units in the polymer chain, but also over the neighbouring chains, further stabilising the charge carrier and promoting its delocalisation. This in turn makes the polymer more susceptible to oxidation and possibly to irreversible oxidative reactions with oxygen.

Through grafting P3HT chains onto the polysiloxane scaffold, we interfere with their ability to organise into lamellae, hindering π–π interactions between the individual chains. This results in slightly elevated band gaps of the copolymers, in relation to **P3HTvin**. Simultaneously, it appears that this hindered organisation of polymer chains increases resistance to the occurrence of noticeable conductance in copolymer films prior to them being electrochemically treated. The resistance appears to be further increased through the incorporation of non-conjugated PEG chains, which also act as a polyelectrolyte, in the presence of ions produced through the dissociation of the supporting electrolyte, possibly assisting in the formation of ‘conductive paths’ in the copolymer films. This process is likely the origin of the changes in the polymer films taking place upon repeated potential cycling at a low potential scanning rate (0.005 V/s).

In part due to this nature of PEG, the grafted copolymers exhibit better doping state reversibility than their parent P3HT polymer. The copolymers also show lower conductivities in the initial undoped state than **P3HTvin**, due to which we can expect them to be less prone to irreversible oxidation, during preparation and their storage, than **P3HTvin**. This is also reflected in the vast improvement of the values of the doped/undoped polymer conductance ratio, with the PEG-bearing copolymers showing ratios on the order of hundreds, while for **P3HTvin** and **P3HT-Sil**, the ratios were below 10.

The incorporation of polysiloxane into the structure of the copolymer appears to have affected its self-organisation properties, increasing the magnitude of effects related to changes in the conformation of the solid state films delivering new promising materials for optoelectronics and photovoltaics. We show preliminary results of BHJ solar cells with efficiency up to 2.46% without optimization demonstrating that new polymers with diluted P3HT chains grafted on polysiloxane backbone can be promising materials for organic photovoltaics.

## Figures and Tables

**Table 1:** Details of the copolymers’ composition. Thiophene units to PEG chains ratio and thiophene units per poly(siloxane) chain were estimated based on ^1^H-NMR analyses

Parameter	P3HT-Sil-PEG1	P3HT-Sil-PEG2	P3HT-Sil-PEG3	P3HT-Sil
Siloxane Mn	390	390	1700–3200	390
P3HT fraction (*M*_n_ by GPC)	hexane (4000)	chloroform (10,000)	chloroform (10,000)	chloroform (10,000)
Fraction of copolymer tested	hexane	chloroform	chloroform	chloroform
PEG co-grafts	Yes	Yes	Yes	No
Thiophene units to PEG chains ratio	112.6	1381.3	2.1	-
Thiophene units per poly(siloxane) chain	12	46	114	14

**Table 2:** Contrasts (given as the absolute value of transmittance differences between the undoped and doped states) achieved for films of varying thickness deposited using the investigated macromolecules, by utilising different types of electrochemical stimuli.

Sample	Undoped Polymer Absorbance	Electrochemical Stimuli	Average Optical Contrast [%]
P3HT	0.18	10 s pulse	14.6
		5 s pulse	13.7
		2 s pulse	13.4
		1 s pulse	13.3
		0.005 V·s^−1^ scan	11.9
	0.36	10 s pulse	13.5
		5 s pulse	13.2
		2 s pulse	13.1
		1 s pulse	12.7
		0.005 V·s^−1^ scan	14.3
P3HT-Sil-PEG1	0.37	10 s pulse	24.8
		5 s pulse	19.0
		2 s pulse	16.7
		1 s pulse	13.9
	0.12	0.005 V·s^−1^ scan	12.2
	0.42	0.005 V·s^−1^ scan	28.1
	0.97	0.005 V·s^−1^ scan	21.1
P3HT-Sil-PEG2	0.33	10 s pulse	22.1
		5 s pulse	20.5
		2 s pulse	19.5
		1 s pulse	18.9
		0.005 V·s^−1^ scan	23.3
	0.71	10 s pulse	11.3
		5 s pulse	8.6
		2 s pulse	7.8
		1 s pulse	7.2
		0.005 V·s^−1^ scan	9.5
P3HT-Sil-PEG3	0.12	10 s pulse	6.2
		5 s pulse	6.2
		2 s pulse	6.1
		1 s pulse	6.1
		0.005 V·s^−1^ scan	6.2
	0.22	10 s pulse	15.6
		5 s pulse	15.5
		2 s pulse	15.4
		1 s pulse	15.4
		0.005 V·s^−1^ scan	15.4
	1.30	10 s pulse	1.6
		5 s pulse	1.5
		2 s pulse	1.0
		1 s pulse	0.7
		0.005 V·s^−1^ scan	2.2
P3HT-Sil	0.13	10 s pulse	4.8
		5 s pulse	4.5
		2 s pulse	4.1
		1 s pulse	3.9
		0.005 V·s^−1^ scan	5.1
	0.04	0.005 V·s^−1^ scan	3.4
	0.27	0.005 V·s^−1^ scan	15.7

**Table 3:** “ON/OFF” conductance ratios of the investigated polymer films.

Polymer/Copolymer	Conductance		Doped/Undoped State Conductance (“ON/OFF”) Ratio
	Undoped State [mS]	Doped State [mS]	
P3HTvin	6.31	20.26	3.21
P3HT-Sil-PEG1	0.10	11.28	112.80
P3HT-Sil-PEG2	0.03	13.61	453.67
P3HT-Sil-PEG3	0.02 (0.51) ^a^	11.44	572.00 (22.43) ^a^
P3HT-Sil	3.19	26.99	8.46

^a^ the values in parentheses are given for the lowest applied potentials rather than the lowest observed conductance.

**Table 4:** Photovoltaic parameters of the BHJ solar cells compared with another copolymers.

Investigated System	*J*_sc_: [mA/cm^2^]	*V*_oc_: [V]	FF:	PCE [%]
P3HT-Sil-PEG/PC_60_BM	8.57	0.63	0.46	2.46
P3HT/PC_60_BM	12.58	0.62	0.45	3.42
P3HT(1.0)-b-PFTBT [38]	4.80	1.15	0.41	2.2
PSB-FTT [39]	9.50	0.82	0.62	4.83
PEGA13/PC61BM [40]	5.80	0.61	0.32	1.1

**Scheme A1:**
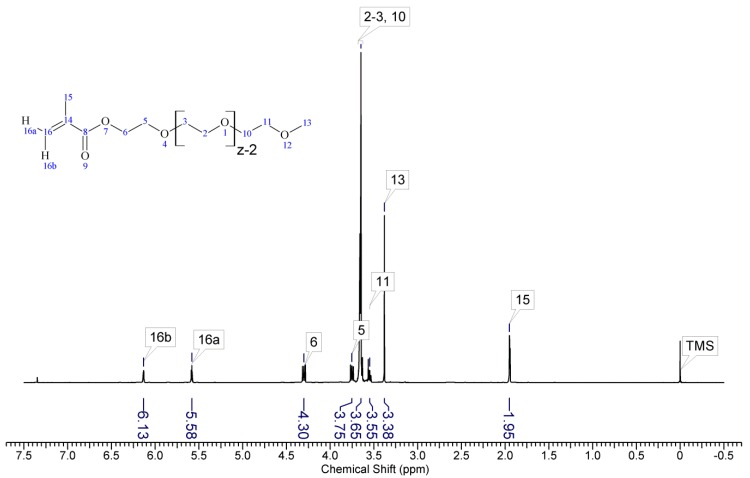
^1^H-NMR spectrum of poly(ethylene glycol) methyl ether methacrylate(z ~ 8).

**Figure A9:**
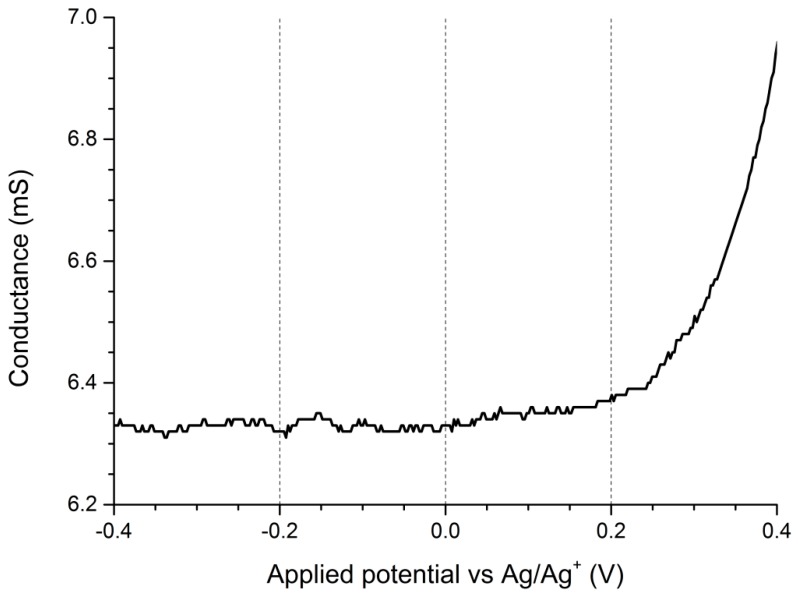
Zoomed in low-potential end of the voltammogram registered for double working electrode electroconductometry of regioregular P3HT film on an interdigitated Pt electrode. Potential offset between the working electrodes Δ*V* = 0.005 V.

## Appendix A

### Appendix A.1. Detailed Synthetic Procedure

**Synthesis of vinyl-terminated regioregular (RR) P3HT (P3HTvin)** via GRIM method: a dry 100 cm^3^ three-necked flask, equipped with septum, a condenser, a gas capillary and a magnetic dipole, was purged with nitrogen and charged via syringe with 2,5-dibromo-3-hexylthiophene (6.13 mmol), anhydrous tetrahydrofuran (50 cm^3^) and t-butylmagnesium chloride (6.13 mmol). The reaction mixture was refluxed for 1.5 h, followed by cooling to room temperature, addition of the Ni(dppp)Cl_2_ catalyst (0.069 mmol) and stirring for the next 1 h. Then vinylmagnesium bromide was added (1.15 mmol) and reaction mixture was stirred for the next 1 h. The crude polymer was precipitated by quenching the reaction mixture in methanol.

**Synthesis of poly(methylhydrosiloxane)-graft-poly(3-hexylthiophene) (P3HT-Sil)**: a dry 100 cm3 three-necked flask, equipped with septum, a condenser, a gas capillary and a magnetic dipole, was charged with vinyl terminated P3HT (0.35 g), purged with nitrogen, charged via syringe with dry toluene (30 cm^3^). After heating to 60 °C and dissolving vinP3HT in toluene poly(methylhydrosiloxane) trimethylsilyl terminated (0.5 cm^3^, PMHS390) and PTDD catalyst (0.03 cm^3^) were added via syringe. Reaction was conducted in 60 °C for 24 h and ended by pouring reaction mixture on Petri dish and evaporating solvent.

**Synthesis of poly(methylhydrosiloxane)-graft-[poly(3-hexylthiophene); poly(ethylene glycol)]s (P3HT-Sil-PEGs)**: a dry 100 cm^3^ three-necked flask, equipped with septum, a condenser, a gas capillary and a magnetic dipole, was charged with vinyl terminated P3HT (0.35 g, hexane fraction – P3HT-Sil-PEG1; chloroform fraction—P3HT-Sil-PEG2 and 3), purged with nitrogen, charged via syringe with dry toluene (30 cm^3^). After heating to 60 °C and dissolving P3HTvin in toluene poly(methylhydrosiloxane) trimethylsilyl terminated (0.1 cm^3^, PMHS390—P3HT-Sil-PEG1 and 2; 0.4 cm^3^, PMHS1700/3200—P3HT-Sil-PEG3) and PTDD catalyst (0.03 cm^3^) were added via syringe. Reaction was conducted in 60 °C for 24 h, then poly(ethylene glycol) methyl ether methacrylate (2.75 cm^3^) was added and reaction mixture was stirred for the next 24 h. Reaction was ended by pouring reaction mixture on Petri dish and evaporating solvent.

### Appendix A.2. Spectra

#### Appendix A.2.1. ^1^H-NMR Spectra:

PEG500 (Scheme A1) ^1^H-NMR (300 MHz, CDCl_3_) δ 6.13 (dd, J = 1.6, 1Hz, 1H)**H**HC=C(CH_3_)–C=O, 5.58 (p, J = 1.6 Hz, 1H)H**H**C=C(CH_3_)–C=O, 4.30 (m, 2H) O=C–O–C**H_2_**–CH_2_–[O–CH_2_–CH_2_–]_n_, 3.75 (m, 2H) O=C–O–CH_2_–C**H_2_**–[O–CH_2_–CH_2_–]_n_, 3.65 (m, 28H)–[O–C**H_2_**–C**H_2_**–]_n_, 3.55 (m, 2H)C**H_2_**–O–CH_3_, 3.38 (s, 3H) o–C**H_3_**, 1.95 (dd, J = 1.6, 1Hz, 3H) =C(C**H_3_**)–C=O.

PMHS390 (Scheme A2) ^1^H-NMR (300 MHz, CDCl_3_) δ4.79–4.62 (m, 9 H) Si–H, 1.58 (s, 1.6 H) water, 0.40–0.05 (m, 63 H) Si–CH_3_.

PMHS1700/32001H ^1^H-NMR (300 MHz, CDCl_3_) δ4.78–4.66 (m, 42H) Si–H, 1.59 (s, 7H) water, 0.40–0.05 (m, 144H) Si–CH_3_.
